# Analyzing collaboration networks and developmental patterns of nano-enabled drug delivery (NEDD) for brain cancer

**DOI:** 10.3762/bjnano.6.169

**Published:** 2015-07-31

**Authors:** Ying Huang, Jing Ma, Alan L Porter, Seokbeom Kwon, Donghua Zhu

**Affiliations:** 1 School of Management and Economics, Beijing Institute of Technology, Beijing 100081, China; 2 Lab of Knowledge Management and Data Analysis (KMDA), Beijing Institute of Technology, Beijing 100081, China; 3 School of Public Policy, Georgia Institute of Technology, Atlanta, GA 30332, USA; 4 Search Technology, Inc., Atlanta, GA 30092, USA

**Keywords:** bibliometrics, brain cancer, collaboration network, nano-enabled drug delivery (NEDD), nanoinformatics

## Abstract

The rapid development of new and emerging science & technologies (NESTs) brings unprecedented challenges, but also opportunities. In this paper, we use bibliometric and social network analyses, at country, institution, and individual levels, to explore the patterns of scientific networking for a key nano area – nano-enabled drug delivery (NEDD). NEDD has successfully been used clinically to modulate drug release and to target particular diseased tissues. The data for this research come from a global compilation of research publication information on NEDD directed at brain cancer. We derive a family of indicators that address multiple facets of research collaboration and knowledge transfer patterns. Results show that: (1) international cooperation is increasing, but networking characteristics change over time; (2) highly productive institutions also lead in influence, as measured by citation to their work, with American institutes leading; (3) research collaboration is dominated by local relationships, with interesting information available from authorship patterns that go well beyond journal impact factors. Results offer useful technical intelligence to help researchers identify potential collaborators and to help inform R&D management and science & innovation policy for such nanotechnologies.

## Introduction

Drug delivery research has grown rapidly over the past two decades and has enabled drug development by designing suitable delivery systems that improve efficacy, lower dosing frequency, and encourage patient convenience and compliance [1]. Within the last ten years, nano-enabled drug delivery (NEDD) has drawn the attention of research and industry areas, as a key nanotechnology. Nanoparticulate drug-delivery vehicles have been developed using various nanomaterials and components (mainly polymers). Such systems have the ability to encapsulate and carry the payload (therapeutics) and penetrate through biological membranes to deliver that payload to specific target disease sites [2–4]. The outstanding advantage of NEDD is that the applicable nanoparticles can keep the pharmaceutical well protected from degradation and prolong the exposure of the pharmaceutical through controlled release. Thus, NEDD provides a novel approach to medical therapy, including treatment of chronic diseases and genetic disorders [5]. At the present, various kinds of nanoparticles have been developed as drug carriers, such as liposomes, micelles, polymeric conjugates and so on [6–8]. Among these, the brain tumor-targeting drug delivery systems, which increase drug accumulation in the tumor region and reduce toxicity in the normal brain and peripheral tissue, are a promising new approach [9].

Collaboration fosters interactions between different actors within and across fields, which reflects sharing of knowledge and other resources [10]. Linkages generated among actors accelerate communication and information exchange for mutual benefit [11]. In these circumstances, research collaboration facilitates keeping up with advances in methods and findings in related fields. It is vital in interdisciplinary arenas and nano-bio-informatics can bolster intelligence concerning advances and potential collaborators. “R&D landscaping” to understand collaboration and developmental patterns can offer global-level insights [12]. This paper aims to support policy-makers or managers who are making strategic technical decisions regarding NEDD for brain cancer gain useful intelligence on technical and international capabilities. The research employs bibliometric, text analytic, and social network analysis methods to explore the collaboration patterns at the country, institution, and author levels to understand better the international development of NEDD for brain cancer.

This paper highlights three points:

1. The international collaboration index (ICI), calculated using a paper collaboration ratio (PCR) and an international collaboration range (ICR), is applied to measure networking for the top 10 countries at the following stages: 1990–1999, 2000–2009 and 2010–2014;
2. An organization diversity index (ODI) and a country diversity index (CDI) are used to judge the collaboration diversity of leading institutions;
3. The matrix of co-authorship performance, which crosses two dimensions – a paper impact index (PII) and an author contribution index (ACI) – locates the contribution of outstanding domain authors.

Together, these provide a new perspective on scientific collaboration and academic evaluation.

The paper is organized as follows: The first section provides general background on NEDD for brain cancer research. In the second part, search strategy and data are introduced. We focus on the scientific activity and collaboration network at the country, institution, and individual levels in the third section. In the conclusion, we make a brief summary of the research findings and identify promising opportunities for further research.

## Search strategy and data

To develop the search strategy of NEDD for brain cancer, we first characterized and classified the essential components, building on a previously developed framework [13].

With the help of knowledgeable colleagues and previous NEDD-related work [14–15], we devised a Boolean, term-based search algorithm for NEDD directed at brain cancer, informed by various reviews and "foresight" pieces. This led us to the following categorization with which to frame our current search, as per Table 1.

**Table 1:** Search Strategy of NEDD for Brain Cancer in Web of Science.

Set	Category	Records	Search Terms

# 1	T (Target)	63,707	TS = (((brain or "central nervous system" or CNS) near/1 (cancer* or anticancer* or tumor* or tumour* or oncology or neoplasm* or carcinoma*)) or glioma* or glioblastoma*)
# 2	N (nanoparticles and materials)	1,135,180	TS = (nano* or micelle* or liposome* or dendrimer* or metal complex* or hydrogel* or “quantum dots*” or chitosan* or alginate*)
# 3	M (Medicine)	128,626	TS = (temozolomide or procarbazine or carmustine or BCNU or lomustine or CCNU or vincristine or everolimus or irinotecan or cisplatin or carboplatin or methotrexate or etoposide or bleomycin or vinblastine or actinomycin or dactinomycin or cyclophosphamide or ifosfamide)
# 4	P (Pharmaceutical)	40,937	TS = (siRNA or "short interfering RNA" or "small interfering RNA")
# 5	D (delivery systems)	4,936,370	TS = (deliver* or vehicle* or carrier* or vector* or treat* or therap* or "control* releas*" or "sustain* releas*" or transduct* or transfect* or transport* or translocat*)
# 6	Final	1859	#1 AND #2 AND (#3 OR #4 OR #5)

We thus obtained 1859 records (language is English and document type is Article), from 1990 to 2014, from the Science Citation Index Expanded (SCI-Expanded) and the Social Sciences Citation Index (SSCI) of the Web of Science (WoS).

**Figure 1:**
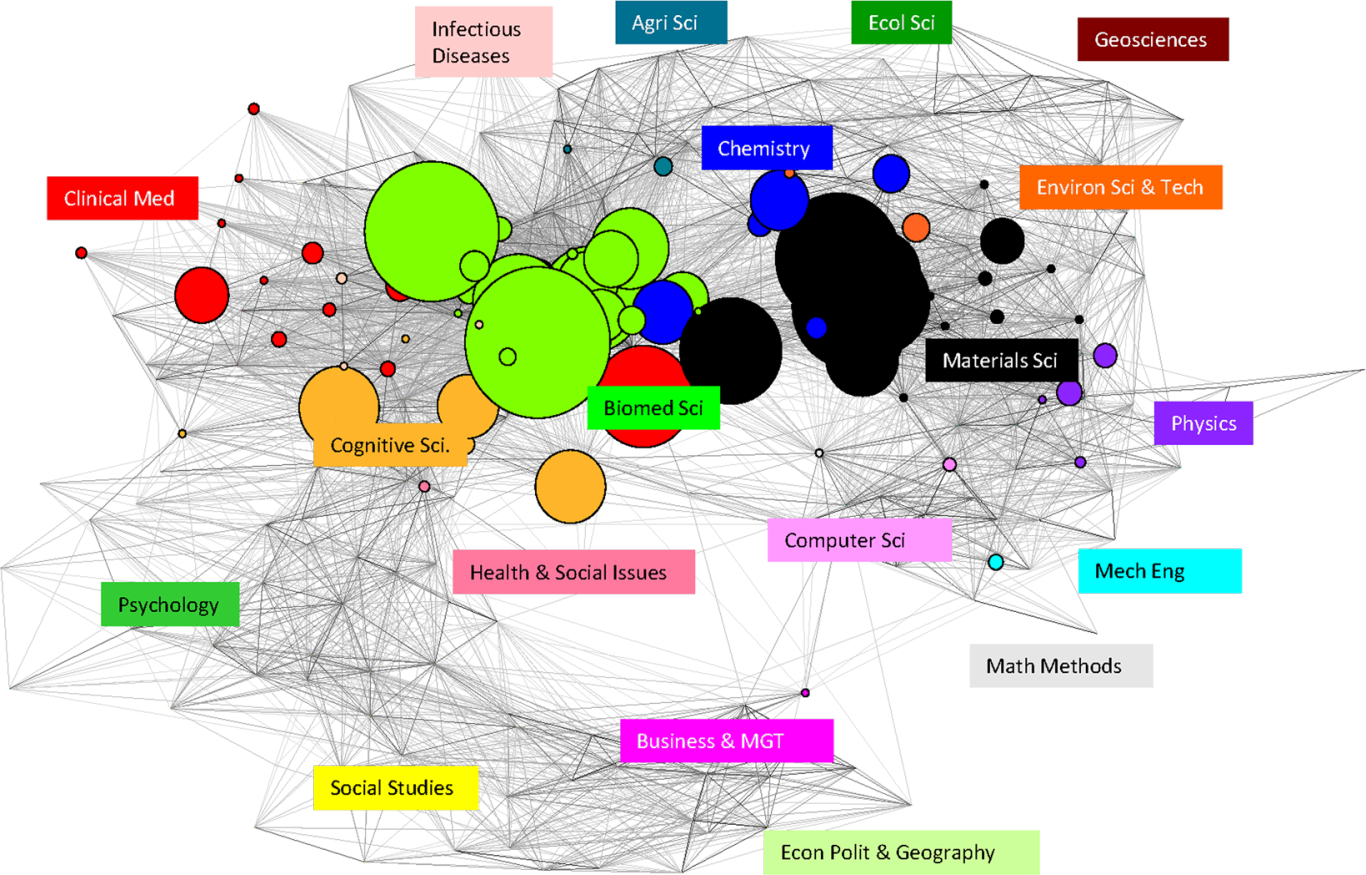
NEDD for brain cancer research across the disciplines.

Nanomedicine research is a multidisciplinary activity, so exploring the disciplinary distribution is illuminating. Figure 1 offers a science overlay map [16] of NEDD for brain cancer, based on the Web of Science categories of the journals in which the 1859 papers appeared. The map shows that biomedicine and materials science are the most active disciplines. Cognitive science, chemistry and clinical medicine are other prominent disciplines.

## Results and Discussion

### International collaboration analysis

**Figure 2:**
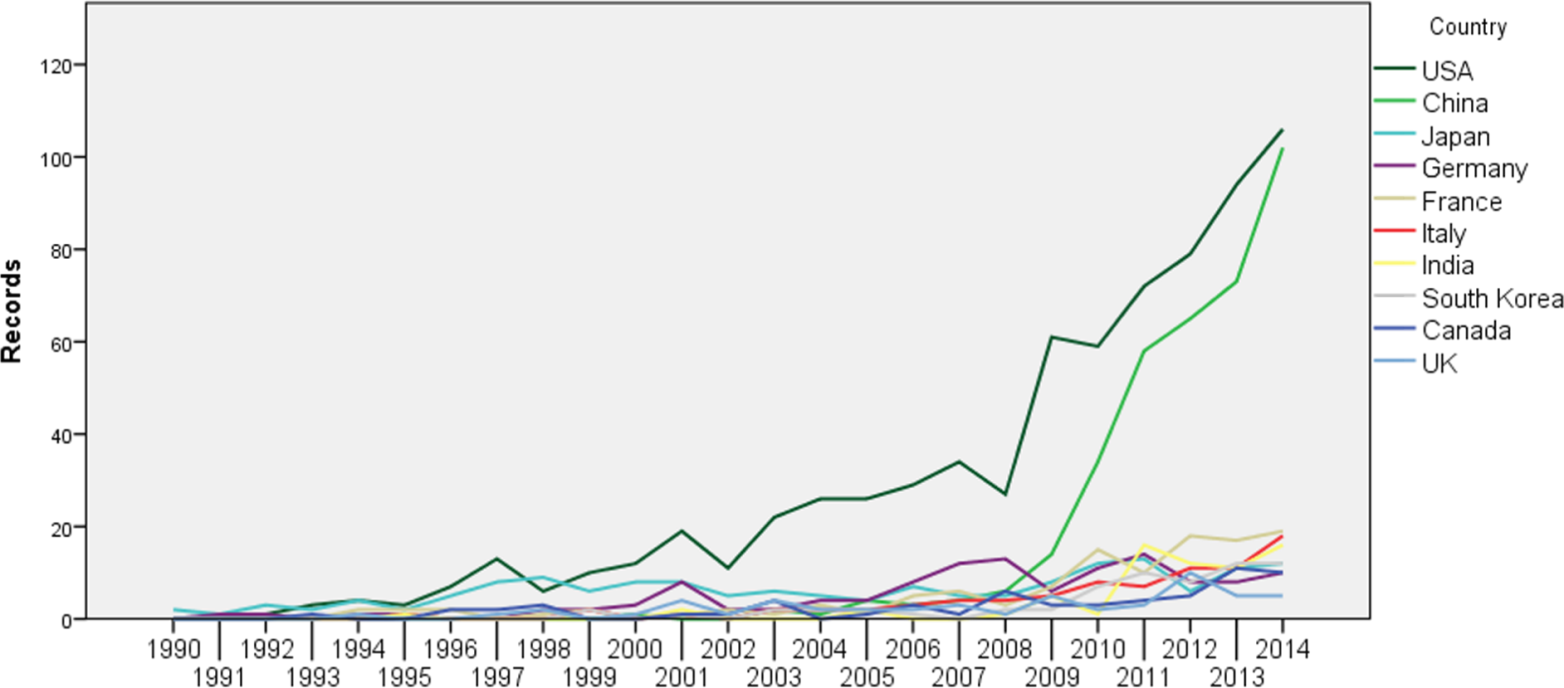
Publication trend of top 10 countries in 1990–2014.

International scientific collaboration has been a driving force for promoting scientific and technological advancement. In this paper we examine the countries of the authors’ affiliations. Figure 2 shows the number of publications by country, based on the location of all author affiliations (not just first authors), from 1990 to 2014.

Among the publication trends, the USA and China stand out. The USA has led over the past 20 years (Japan had a small advantage in 1998), yet China has dramatically caught up over the last 5 years. According to this trend, China will boast the largest proportion of literature in the near future, and the USA and China will remain the key players in the field of NEDD for brain cancer.

To better understand the various development patterns of the top 10 countries, we introduce centrality analysis models that help answer the question, "What characterizes an important vertex?” [17]. These models are degree centrality (DC), closeness centrality (CC), and betweenness centrality (BC).

For DC, which is defined as the number of links incident upon a node, the USA maintains the highest value, meaning that US researchers have more linkages with researchers in other countries. Germany also has wide academic collaboration networks, especially since 2000.

Based on CC, which is a measure of the total distance to sequentially spread information to all other nodes [18], the USA is located in the core position, making it more likely to collaborate with other countries. All other countries share a similar distance among other nodes, from 2000 to 2014.

From the BC perspective, the USA and Germany perform well, acting as a bridge along the shortest path between two other countries. The most striking finding is that, although China is a leader in publication, it plays a quite limited role in connecting other countries (shown as Table 2).

**Table 2:** Centrality analysis for top 10 countries in different stages.

	1990–1999			2000–2009			2010–2014		

	DC	CC	BC	DC	CC	BC	DC	CC	BC

USA	8	0.833	0.208	23	0.706	0.340	35	0.709	0.392
China	0	0.000	0.000	6	0.486	0.011	11	0.519	0.030
Japan	1	0.476	0.000	7	0.522	0.044	11	0.514	0.057
Germany	3	0.588	0.084	16	0.643	0.225	21	0.596	0.099
France	2	0.500	0.000	7	0.522	0.054	19	0.554	0.115
Italy	0	0.000	0.000	5	0.468	0.006	18	0.583	0.091
India	0	0.000	0.000	3	0.439	0.001	11	0.500	0.017
South Korea	0	0.000	0.000	1	0.419	0.000	10	0.519	0.015
Canada	3	0.526	0.003	5	0.500	0.003	12	0.533	0.058
UK	1	0.476	0.000	13	0.581	0.139	11	0.519	0.021

**Figure 3:**
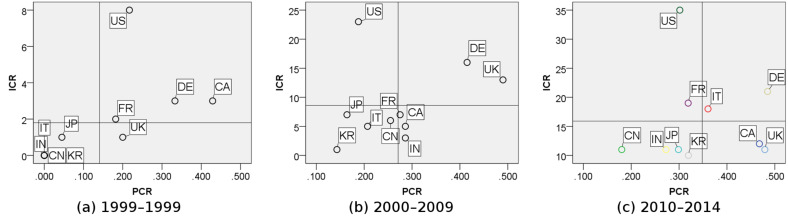
Scatter plot of international collaboration index (ICI) for top 10 countries in (a) 1990–1999; (b) 2000–2009; (c) 2010–2014. The guides indicate the average value of these top 10 countries.

Additionally, the international collaboration index (ICI), calculated by paper collaboration ratio (PCR) and international collaboration range (ICR), is applied to measure the top academic internationalization degree for the top 10 countries within 1990–1999, 2000–2009 and 2010–2014 respectively, as shown in Figure 3.

(1) Paper collaboration ratio (PCR) is defined as how much a country’s multinational papers accounted for the country or region’s total number of papers. This is derived from “the share of international publications” [19].

[1]
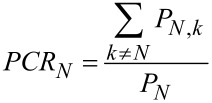


In Equation 1, *N* indicates the country want to calculate, *P_N,k_* is the number of papers produced from the cooperation between country ‘*N*’ and country ‘*k*’. Thus,


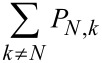


represents the total amount of multinational papers produced from a certain country or region that has taken part in related research by collaboration with the country ‘*N*,’ and *P_N_* represents the total amount of papers produced from the country ‘*N*’.

(2) International collaboration range (ICR) is defined as how many partner countries have been involved in collaborations and reflects the breadth of one country or region’s international collaboration from a macro view [20].

[2]
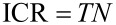


In Equation 2, *TN* is the total number of countries or regions with which a country or region has cooperated.

From the scatter plots (Figure 3), we identify some interesting findings:

1. All the top 10 countries show an improvement, both in PCR and ICR, which indicates that international cooperation is becoming more and more frequent in the field of NEDD for brain cancer;
2. The USA always leads the global research, and it has the widest academic collaboration networks and relatively fruitful cooperation outcomes;
3. Compared to other regions, Asian countries, including China, Japan, India and South Korea, are located at the low-ICR and low-PCR area, which means they have relatively less connection with researchers of other nationalities, despite their recent growth in articles published.

### Institutional co-authorship analysis

**Figure 4:**
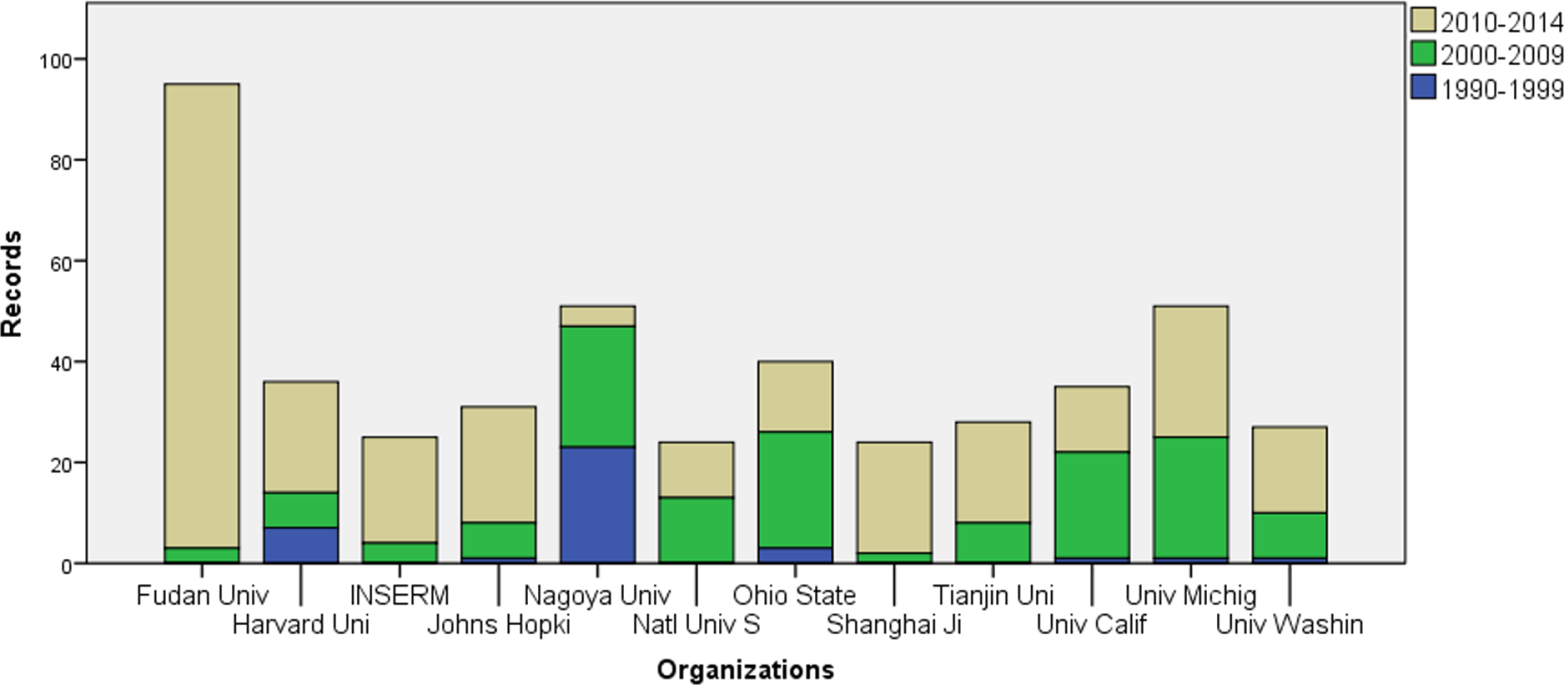
Publications of the top 12 institutions in 1990–2014.

In general, the research levels of a certain country depend on its leading institutions. Figure 4 shows the 12 leading institutions in NEDD for brain cancer research. Most institutions show good performance for the last 5 years, and Fudan University achieves an amazing number of research results, showing that their number of publications between 2010 and 2014 is far greater than any other institution in the same time period. Nagoya University led the domain development previously, but it encountered a serious decline recently and is losing ground.

Among these top 12 institutions, half come from the USA, three are from China, and the remaining three organizations are in Japan, France, and Singapore. Citations that establish links to other works or other researchers are treated as an indicator of impact [21]. From Table 3, we can see that papers published by the National University of Singapore are the most cited by other researchers (63 per paper), and they also reference more previous work (57 records) per publication. Additionally, some other institutions from the USA perform outstandingly in citations as well, including the University of Michigan, Harvard University, and the University of Washington. However, citation is usually skewed, so we introduce the median times cited that is the median value of all times cited to further evaluate the citation behavior. University of California, San Francisco (UCSF) shows most expressive performance in median times cited, and followed by University of Michigan, Ohio State University and National University of Singapore. Even through Harvard University stands out in average times cited, most of the citations are contributed by the few highly cited papers.

**Table 3:** Publications and citation information for the top 12 institutions.^a^

Organization	Records	Average times cited	Median times cited	Average times citing	Country

Fudan Univ	95	19.9	12.0	13.0	China
Nagoya Univ	53	25.5	14.0	17.0	Japan
Univ Michigan	51	52.9	27.0	38.3	USA
Ohio State Univ	40	35.5	27.0	24.0	USA
Harvard Univ	37	52.0	19.5	46.4	USA
Univ Calif San Francisco	35	38.7	32.0	23.9	USA
Johns Hopkins Univ	31	24.6	10.0	23.1	USA
Tianjin Univ	28	32.0	14.0	26.6	China
Univ Washington	27	47.3	21.0	35.5	USA
INSERM	24	13.8	7.5	11.8	France
Natl Univ Singapore	24	63.2	26.5	57.4	Singapore
Shanghai Jiao Tong Univ	24	13.2	3.0	10.7	China

^a^Citation counts include self-citation.

In the area of collaboration activity, we introduce the organization diversity index (ODI) and the country diversity index (CDI) to locate the top 12 institutions.

(1) ODI is defined as the index of the collaboration distributions and collaboration times of certain organizations with other organizations through multi-institutional publications. It can be expressed as follows:

[3]
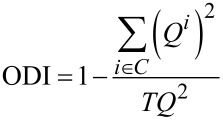


In Equation 3, *Q^i^* represents the number of multi-institutional publications involving collaborators from certain institutions '*i*'. *C* represents the set of historical collaborators of the targeted organization, *TQ* represents the total multi-institutional publications of the organization.

(2) CDI has a definition similar to ODI, but it is set to explore the country level, rather than the institutional level.

**Figure 5:**
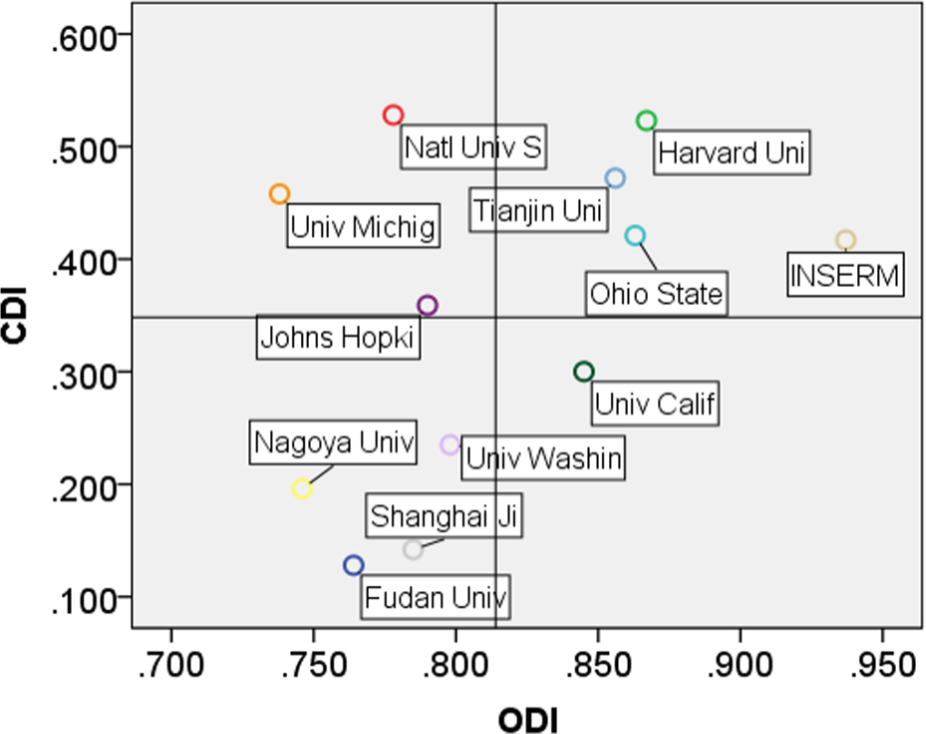
Collaboration activity of the top 12 institutions.

In Figure 5 we see that Harvard University, INSERM, Tianjin University, and Ohio State University have wide international academic collaboration and influential research results. In comparison with some other institutions, such as University of Michigan and National University of Singapore, they tend to have more connections with international institutions than domestic organizations. The University of California, San Francisco (UCSF), has a strong partnership with other institutions in its country. Other leading institutions – including Fudan University, Nagoya University, Johns Hopkins University, and the University of Washington – have strong cooperative relations inside their organizations.

### Author activity analysis

**Figure 6:**
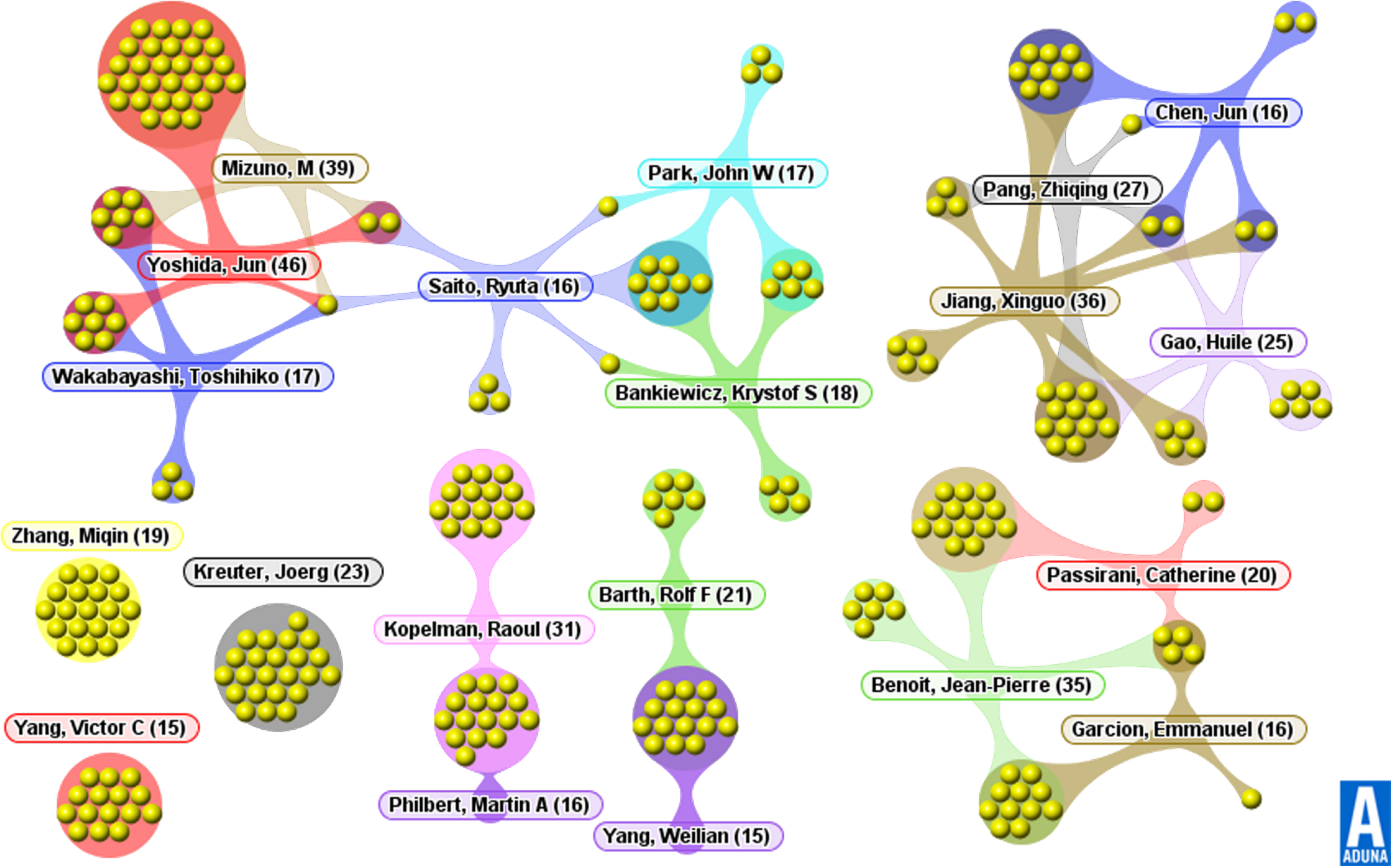
Co-author network of the top 20 authors.

No matter the advantages of a country or the influence of an institution, it is the researchers that make them truly great. Exploring the core authors in the NEDD for brain cancer field can help researchers take advantage of leading potential cooperative partners. Figure 6 shows the co-author network of the top 20 authors, in terms of numbers of research papers. From Figure 6, we see that the majority of the authors in the NEDD for brain cancer field have strong connections in the micro-community. In other words, they often come from the same institution (see Table 4). There are five main partnering relationships: Nagoya University–University of California, San Francisco (UCSF) group, Fudan University group, University of Angers group, Ohio State University group, and a University of Michigan group.

Even though the USA ranks first in this new field, none of its authors rank in the top three of the author list, and only two rank in the top 10. Yoshida and Mizuno, both of whom come from the Department of Neurosurgery, Nagoya University School of Medicine in Japan, rank first and second in the list, respectively. Among the top 20 authors, however, US scholars represent 45% of the total, showing that the USA does hold a strong position in NEDD brain cancer research. Besides the representation from the USA, four authors come from China, three from France, three from Japan and one from Germany.

It should be noted that authorship analysis focuses on the productivity of authors and their contributions in their respective fields. In multi-authored papers, the first author position is occupied by the individual nominally making the greatest contribution [22]. Authors in the top 20 list, while productive in domain publications, are not often listed as the first author. Only 4 researchers occupy the first position in more than 20% of their respective papers. In addition, publication amounts do not always match the results of the citation evaluation, which can be observed as the average times cited and the h-index (shown as Table 4). What’s more, we can figure out that the research areas of these leading researchers tend to emphasize oncology, materials science, pharmacology & pharmacy, chemistry, neurosciences & neurology, research & experimental medicine and engineering, which indicates that nanomedicine research is a multidisciplinary activity. At the same time, researchers coming from the same institution tend to focus on similar research areas and collaborate on within-domain research.

**Table 4:** Top 20 authors in NEDD for brain cancer.

Authors	Records	1st-Author records	Average citations	h-index	Organization	Country	Research area

Yoshida, Jun	46	7	24.59	18	Nagoya Univ	Japan	Oncology; Neurosciences & Neurology
Mizuno, M	39	8	18.74	15	Nagoya Univ	Japan	Oncology; Neurosciences & Neurology
Jiang, Xinguo	36	0	21.36	14	Fudan Univ	China	Materials Science; Pharmacology & Pharmacy
Benoit, Jean-Pierre	35	0	16.4	13	Univ Angers	France	Pharmacology & Pharmacy; Chemistry
Kopelman, Raoul	31	1	61.97	19	Univ Michigan	USA	Chemistry; Materials Science
Pang, Zhiqing	27	2	22.71	14	Fudan Univ	China	Materials Science; Pharmacology & Pharmacy
Gao, Huile	25	14	12.81	9	Fudan Univ	China	Pharmacology & Pharmacy; Materials Science
Kreuter, Joerg	23	3	68.26	18	Univ Frankfurt	Germany	Pharmacology & Pharmacy; Chemistry
Barth, Rolf F	21	3	51.9	17	Ohio State Univ	USA	Oncology; Chemistry
Passirani, Catherine	20	0	15.5	10	Univ Angers	France	Pharmacology & Pharmacy; Chemistry
Zhang, Miqin	19	0	53.68	13	Univ Washington	USA	Materials Science; Chemistry
Bankiewicz, Krystof S	18	0	38.35	13	Univ Calif San Francisco	USA	Neurosciences & Neurology; Oncology
Park, John W	17	1	53.83	14	Univ Calif San Francisco	USA	Neurosciences & Neurology; Oncology
Wakabayashi, Toshihiko	17	2	32.24	11	Nagoya Univ	Japan	Oncology; Research & Experimental Medicine
Chen, Jun	16	0	18.48	11	Fudan Univ	China	Materials Science; Enginerring
Garcion, Emmanuel	16	1	19.56	9	Univ Angers	France	Pharmacology & Pharmacy; Chemistry
Philbert, Martin A	16	0	86.25	15	Univ Michigan	USA	Chemistry; Pharmacology & Pharmacy
Saito, Ryuta	16	7	47.81	11	Univ Calif San Francisco	USA	Neurosciences & Neurology; Oncology
Yang, Victor C	15	0	48.73	12	Univ Michigan	USA	Materials Science; Pharmacology & Pharmacy
Yang, Weilian	15	6	13.6	8	Ohio State Univ	USA	Oncology; Chemistry

In order to better evaluate author activity, we introduce the paper impact index (PII) and author contribution index (ACI).

(1) Paper impact index (PII) is defined as the sum of the impact factors of all published papers. It can be expressed as follows:

[4]
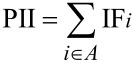


In Equation 4, IF*_i_* represents the impact factor (IF) of the journal that published the article ‘*i*’ of certain author, as indicated by the journal citation reports (JCR), provided by Thomson Reuters; ‘*A*’ represents the set of articles that the author published.

(2) Author contribution index (ACI) is defined as the total contribution of the author in all authored papers. Authorship order only reflects relative contribution (with considerable variability in norms), whereas evaluation committees often prefer other quantitative measures. A reasonable method for quantifying contributions is to give the first author credit for the whole contribution, the second author half, the third a third, and so forth [23]. In this paper, we take the value as follows:

[5]
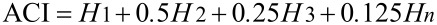


In Equation 5, *H*_1_, *H*_2_, *H*_3_ represents the number of a certain author’s first-, second- and third-author papers within a period, and *H_n_* represents the number of papers in which his or her name appears after the first three in the authorship order.

**Figure 7:**
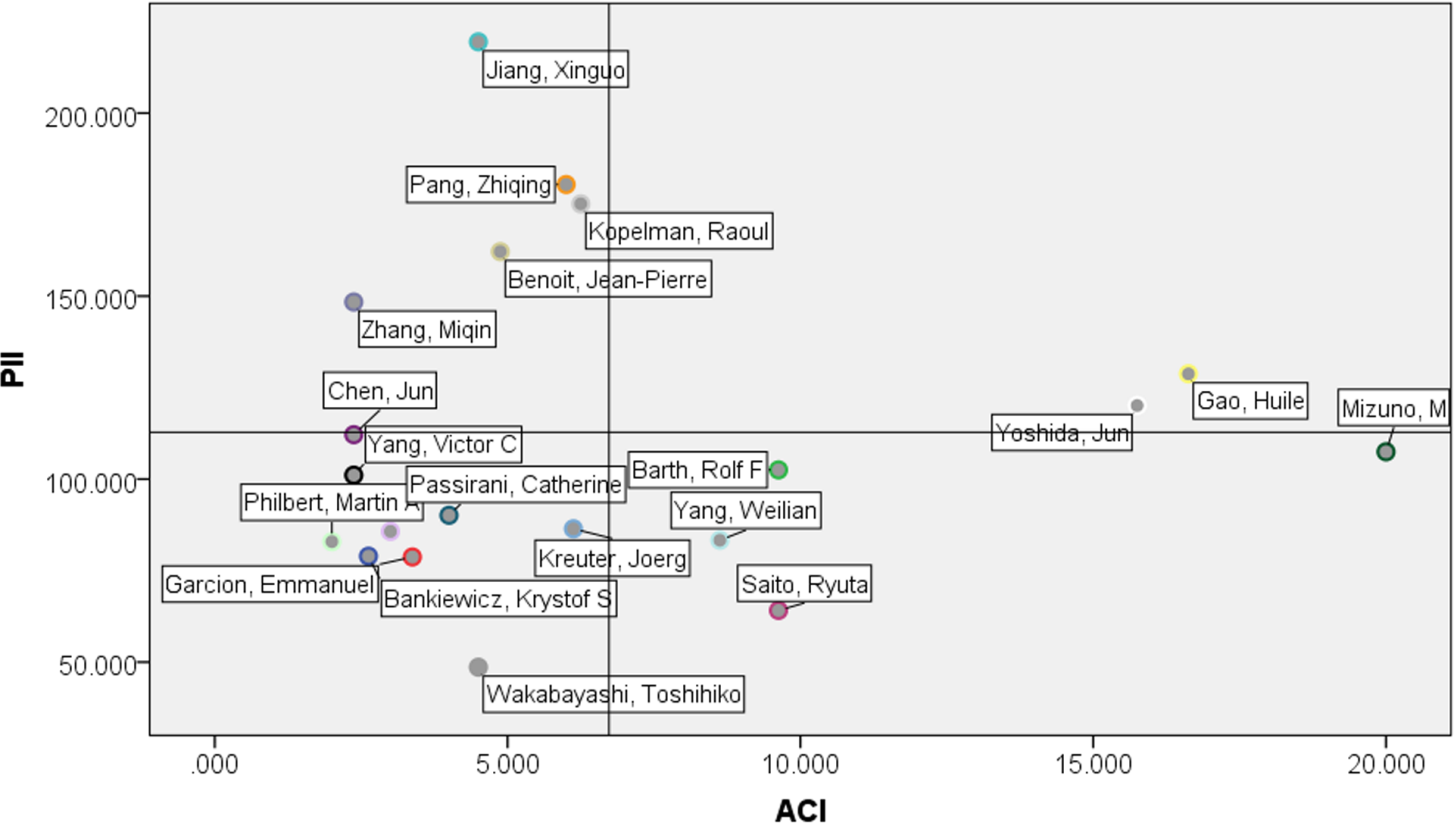
The author activities of top 20 authors in NEDD for brain cancer.

The author activities of top 20 authors in NEDD for brain cancer are shown as Figure 7.

From the standpoint of research performance, many authors publish papers in high IF journals, which allows their work to be more widely accessible and more influential on other researchers. Jiang (Fudan University), Pang (Fudan University), Kopelman (University of Michigan), Benoit (University of Angers), and Zhang (University of Washington) have similar activity patterns of marked research influence. From the standpoint of contributions in multi-authored publications, Mizuno (Nagoya University), Yoshida (Nagoya University) and Gao (Fudan University) all published more papers as the first author during our survey period.

Typically, advanced scholars will publish their research results in high IF journals, while promising scholars publish more papers as the first author. According to this logic, the author activity pattern can be divided into four types, based on academic influence and research contribution:

1. High-PII and High-ACI: Prestigious and active researchers
2. High-PII and Low-ACI: Experienced and senior researchers
3. Low-PII and High ACI: Growing and promising researchers
4. Low-PII and Low-ACI: New and emerging researchers.

Thus, we see that, in the NEDD for brain cancer field, there is a leading minority of key authors while most of the other authors are still in the stage of exploring this NESTs.

## Conclusion

The above analyses reveal some interesting and meaningful findings for the NEDD for brain cancer field:

1. International cooperation is becoming more and more frequent overall, but most countries have different cooperation characteristics, and their academic status varies in different periods.
2. Leading institutes with higher publication numbers perform strongly in terms of citations. American institutes are especially prominent, both in citation behavior and in the collaboration index, as measured by country diversity and organization diversity.
3. Academic researchers tend to seek internal partnerships. Their contributions in published literature should be further evaluated with respect to authorship patterns, even though these publications are accepted by high-impact journals.

NEDD systems are rapidly growing as a key area for nanotechnology application and emerging on a variety of R&D fronts to address a large range of challenges, and curing brain cancer is a high potential application of NEDD that is worth of more exploration. Exploring nano biomedicine research from the respective of social science causes us great interest. Literature informatics, such as our multi-tier R&D landscaping, can help inform science policy makers about collaboration patterns and help technology managers prioritize developmental prospects. Analyzing large compilations of research publication (and/or patent) records can help track developmental trajectories and forecast innovation pathways. Topical analyses within field, not emphasized here, can further aid researchers in identifying potentially useful techniques and research findings in adjacent fields, as well as spotting potential collaborators. The method proposed in the paper can be applied to other research fields to support policy-makers or managers who are making strategic technical decisions with the goal to enhance their technological innovation capabilities and international competitiveness.
